# Advancing patient evidence in XLH (APEX): rationale and design of a real-world XLH global data unification program

**DOI:** 10.3389/fendo.2025.1471127

**Published:** 2025-04-07

**Authors:** Maria Luisa Brandi, Thomas O. Carpenter, Seiji Fukumoto, Dieter Haffner, Erik A. Imel, Masanori Kanematsu, Keith P. McCullough, Keiichi Ozono

**Affiliations:** ^1^ Fondazione Italiana Ricerca sulle Malattie dell'Osso (FIRMO), Florence, Italy; ^2^ Department of Pediatrics, Section of Endocrinology, Yale School of Medicine, New Haven, CT, United States; ^3^ Tamaki - Aozora Hospital, Tokushima, Japan; ^4^ Department for Pediatric Kidney, Liver, Metabolic and Neurological Diseases, Hannover Medical School, Hannover, Germany; ^5^ Departments of Medicine and Pediatrics, Indiana University School of Medicine, Indianapolis, IN, United States; ^6^ Kyowa Kirin Corporation, Tokyo, Japan; ^7^ Arbor Research Collaborative for Health, Ann Arbor, MI, United States; ^8^ ISEIKAI International General Hospital, Osaka, Japan

**Keywords:** X-linked hypophosphatemia (XLH), *phosphate-regulating endopeptidase homolog* *X-linked (PHEX)* gene, fibroblast growth factor 23 (FGF23), musculoskeletal, rickets, osteomalacia, odontomalacia, Advancing Patient Evidence in XLH (APEX)

## Abstract

X-linked hypophosphatemia (XLH) is a rare, genetic, progressive, lifelong disorder caused by pathogenic variants in the *phosphate-regulating endopeptidase homolog, X-linked (PHEX)* gene, resulting in excess fibroblast growth factor 23 (FGF23) and consequent renal phosphate wasting. Chronic hypophosphatemia leads to deficits of the musculoskeletal system affecting bone, muscle, joint, and dental health. XLH treatments include oral phosphate and active vitamin D—which are associated with a burdensome dosing regimen, gastrointestinal disturbances, hyperparathyroidism, and nephrocalcinosis—or burosumab, a fully human anti-FGF23 antibody. Randomized clinical trials (RCTs) demonstrated burosumab to be well tolerated and efficacious in improving serum phosphate, rickets, bone turnover, and patient-reported outcomes. However, there are limited data on the natural history of XLH or real-world comparisons of the safety, effectiveness, and long-term outcomes of XLH treatments. Advancing Patient Evidence in XLH (APEX) is a global data unification project aiming to describe the burden and lifelong progression of XLH, collect real-world data on treatment effectiveness and safety, and investigate regional differences in treatment outcomes. Participants from three observational, noninterventional, retrospective and prospective, multicenter, longitudinal (10-year) studies of patients with XLH will be included: XLH Disease Monitoring Program (NCT03651505), International XLH Registry (NCT03193476), and SUNFLOWER (NCT03745521). Data collected in the Americas, Europe, Israel, Japan, and South Korea will be processed to unify identical and similar data elements. Data unification will be an iterative process with a clinical and programming review, ensuring validity and accuracy. In this observational study, unified data involving approximately 2000 pediatric and adult participants with XLH will be analyzed to address research questions in an exploratory manner. Long-term observational studies and patient registries provide opportunities to generate real-world data and address knowledge gaps in rare diseases. APEX aims to improve clinical decision-making and practice by bridging evidence gaps that cannot be addressed by RCTs or regional registries.

## 1 Introduction

X-linked hypophosphatemia (XLH) is a rare, progressive and lifelong disorder caused by pathogenic variants in the *phosphate-regulating endopeptidase homolog, X-linked (PHEX)* gene, resulting in excess fibroblast growth factor 23 (FGF23) and consequent renal phosphate wasting (1–3) affecting approximately 1 in 20,000 to 70,000 people globally (4–7). XLH is caused by inactivating *PHEX* variants (1, 8) and is inherited in an X-linked dominant pattern (8). *De novo* variants occur in approximately 20% to 30% of patients (9, 10). Increased levels of FGF23 cause downregulation of the sodium-dependent phosphate co-transporters NPT2a and NPT2c in proximal renal tubules leading to renal phosphate wasting, decreased 1α-hydroxylase, and increased 24-hydroxylase activity, resulting in decreased serum 1,25-dihydroxyvitamin D levels, and decreased phosphate absorption in the intestines (1). Resultant chronic hypophosphatemia impairs bone and tooth mineralization resulting in rickets, osteomalacia, and odontomalacia (1, 8), amongst other multi-system consequences. In children, impaired and disproportionate growth with lower limb deformities are typical; delayed motor development and craniosynostosis may occur (1, 8, 11, 12). Adults with XLH may manifest fractures or pseudofractures, early onset osteoarthritis, enthesopathy, and less frequently, spinal stenosis (1, 8, 11). Hearing loss is not infrequent (1, 8). Dental abscesses are common across age groups as well as bone and joint pain, stiffness, and muscle weakness (1, 8, 11). The disorder severely impacts physical function, mobility, and health-related quality of life (QoL) (13, 14). In addition to the well characterized role of elevated FGF23 and hypophosphatemia in XLH, inactivating *PHEX* variants also modify levels of the small integrin-binding ligand, N-linked glycoproteins (SIBLING) family, such as osteopontin and the inhibitory acidic serine aspartate-rich-MEPE-associated protein (ASARM) peptides (1, 15–17). These lead to local impairment of mineralization of bone and teeth, and altered tooth morphology, contributing to osteomalacia and odontomalacia in XLH (1, 15–17). Efforts to treat XLH medically have focused primarily on modifying the influence of FGF23 on renal phosphate handling and 1,25-dihydroxyvitamin D production, or administration of conventional therapy, neither of which are likely to address aspects of the disease that are not mediated by FGF23, hypophosphatemia, or impaired vitamin D activation.

“Conventional therapy” over the past several decades has consisted of a combination of oral phosphate salts and active vitamin D analogs (8, 18). While this treatment is usually beneficial, it does not address the elevated levels of FGF23, and phosphate levels cannot be safely normalized (8, 18). Furthermore, conventional therapy does not normalize growth, nor entirely resolve bone and dental manifestations, or improve health-related QoL (13, 14, 19–21). Additionally, significant side effects, such as secondary or tertiary hyperparathyroidism, hypercalciuria, and nephrocalcinosis, may occur, and progression to chronic kidney disease has been described (18). Finally, oral phosphate supplementation is associated with poor adherence due to its unpleasant taste, the burdensome regimen of frequent dosing, and uncomfortable side effects such as diarrhea and abdominal cramping (8, 22, 23).

Burosumab is a fully human monoclonal antibody against FGF23, approved initially in 2018 for the treatment of children and adults with XLH, with varied approvals and payor coverage in children and adults across the world (24–26). Unlike conventional therapy, burosumab targets an earlier stage in the underlying pathophysiology of XLH by blocking effects of excess FGF23 and improving serum phosphate levels (24, 25, 27, 28). Clinical trials have shown burosumab to improve serum phosphate levels without hypercalciuria or elevating parathyroid hormone levels (29, 30). In children, burosumab improved rickets, and increased height and walking distance in the 6-Minute Walk Test (30). In adults, insufficiency fractures healed, walking distance in the 6-Minute Walk Test improved, and patient-reported outcomes of stiffness, pain, physical function improved (31). These benefits of burosumab treatment have been shown to be maintained long-term, for up to 144 to 184 weeks in adults (32, 33) and up to 160 weeks in children (34). Burosumab was well tolerated in clinical trials with few, if any, treatment-related serious adverse events (SAEs) (29–31, 35). Restless leg syndrome was observed as an adverse event (AE), primarily in adults (35). There were no AEs or SAEs, including deaths, that led to withdrawal of participants from the studies (29–31, 35). Neutralizing antibodies against burosumab were infrequently detected (29–31, 35, 36) and patients positive for neutralizing antibodies responded to burosumab treatment (36). To date, burosumab has not been found to increase nephrocalcinosis, ectopic myocardial mineralization, or incidence of hyperparathyroidism. However, there are important knowledge gaps regarding the long-term effects of burosumab treatment.

One such knowledge gap is the expected inability of burosumab to treat manifestations of XLH that are mediated by factors other than FGF23, such as osteopontin and ASARM peptides. To date, the effect of burosumab is not known regarding final height, degree of improvement of lower limb deformities, craniosynostosis, or the long-term effects regarding dental complications in patients with XLH (27, 30, 37–49). However, it is important to consider that most of these patients were previously treated with conventional therapy prior to burosumab initiation, and the age of burosumab initiation varied, which may influence the cumulative development of disease-related complications. Outside of randomized clinical trials (RCTs), burosumab was able to improve or prevent the worsening of some of these complications (27, 38–49), and earlier age of initiation improved outcomes relative to later age of initiation (42, 47, 49). Additionally, patients who initiate burosumab treatment tend to be those who exhibit more severe symptomatology compared with patients who persist in treatment with conventional therapy (43, 46). Therefore, information on the effect of long-term burosumab treatment and the impact of the age of initiation will be important in understanding the role burosumab can play in treating the wider symptomatology of XLH and, in addition, will also provide insights into which disease features cannot be addressed by targeting FGF23.

Due to the rarity of XLH, and with treatment expertise limited to specialized centers, significant unmet needs exist for affected patients (8). Real-world evidence is required to address knowledge gaps and to provide further information on the natural history of XLH, disease progression and burden, as well as long-term effectiveness, safety and the overall experience with burosumab outside of clinical trial conditions (2). Patient registries have been shown to be effective in the collection of large-scale, real-world patient data in rare disorders (50).

The Advancing Patient Evidence in XLH (APEX) is a ten-year global data unification project, combining data from three Kyowa Kirin-/Ultragenyx-sponsored regional observational studies to increase the overall size of the datasets and address a set of questions that would not be feasible or complete using the regional studies alone.

## 2 Methods

### 2.1 Regional study identification

Three Kyowa Kirin-/Ultragenyx-sponsored, regional observational studies in patients with XLH have been established and registered with ClinicalTrials.gov (**Table 1**). The XLH Disease Monitoring Program (XLH DMP, NCT03651505), initiated in July 2018 and with enrollment completed in December 2022, collects data in the Americas (51). The International XLH Registry (IXLHR, NCT03193476), collecting data from Europe and Israel, was initiated in September 2017 (52). The Study of Longitudinal Observation for Patients With X-linked Hypophosphatemic Rickets/Osteomalacia in Collaboration With Asian Partners (SUNFLOWER, NCT03745521), collecting data from participants in Japan and Korea, was initiated in April 2018 and with enrollment completed in April 2022 (53).

**Table 1:** Characteristics of Kyowa Kirin-/Ultragenyx-sponsored regional XLH studies.

Registry	Country	Sponsor	Study Type	Number of Patients*	Patient Age	Number of sites	Study Period
XLH DMP	US, Canada, and Latin America	Ultragenyx, Kyowa Kirin North America	Company-sponsored, prospective, multicenter observational, phase IV study for XLH	776, enrollment completed Dec 2022	All ages	35	Jul 2018–Jul 2028 (10 years)
IXLHR	Europe, Israel; 21 countries^†^	Kyowa Kirin International	Company-sponsored, prospective, multicenter, non-interventional observational registry	1257, as of Apr 15, 2024	All ages	121	Sep 2017–Dec 2028 (10 years)
SUNFLOWER	Japan, Korea	Kyowa Kirin Co., Ltd.	Company-sponsored, prospective, observational cohort study	226, enrollment completed Apr 2022	All ages	20	Apr 2018–Dec 2028 (10 years)

*Not all participants enrolled at the regional studies are included in the APEX study, depending on data availability and participant consent. ^†^Countries participating in the IXLHR: Belgium, Bulgaria, Czech Republic, Denmark, France, Germany, Hungary, Ireland, Israel, Italy, Latvia, The Netherlands, Norway, Portugal, Romania, Slovakia, Slovenia, Spain, Sweden, Switzerland, United Kingdom. DMP, Disease Monitoring Program; IXLHR, International XLH Registry; SUNFLOWER, Study of Longitudinal Observation for Patients With X-linked Hypophosphatemic Rickets/Osteomalacia in Collaboration With Asian Partners; XLH, X-linked hypophosphatemia.

### 2.2 Inclusion and exclusion criteria

Inclusion and exclusion criteria for the three regional studies are described in **Table 2**. Briefly, patients diagnosed with XLH who provided consent and were not participating in a clinical trial at the time (or who had received prior approval to do so in the case of the XLH DMP) were eligible for participation in the individual studies (2, 51–54). Patients were identified and enrolled by participating clinical sites.

**Table 2:** Inclusion/exclusion criteria of Kyowa Kirin-sponsored regional XLH studies.

Inclusion/Exclusion	Criteria	XLH DMP	IXLHR	SUNFLOWER
Inclusion criteria	Age and sex	Patients of all ages at baseline		Patients of any age who have been clinically diagnosed with XLH
	XLH diagnosis	Clinical diagnosis of XLH based on clinical features including short stature or leg deformities AND biochemical profile consistent with XLH, OR confirmed *PHEX* mutation in patient or in family member	Diagnosis of XLH with clinical, radiological, biochemical, genetic AND/OR family mapping findings consistent with XLH	Patients have one or more of the following: a documented *PHEX* mutation, at least one family member with a documented *PHEX* mutation or a documented intact FGF23 level >30 pg/mL (cut-off value for diagnosing FGF23-related hypophosphatemia)
	Symptomatology	Not defined		Participants must also have a history or current physical signs of rickets/osteomalacia, regardless of treatment history (presence/absence of treatment or type of treatment)*
	Consent	Participants must provide written informed consent/assent		
Exclusion criteria	Consent	Patient or their legally authorized representative is not willing and able to provide informed consent after the nature of the study has been explained and prior to any study procedures	Patient or their legally designated representative does not have the cognitive capacity to provide informed consent	Not defined
	Clinical trial participation	Concurrent enrollment in a clinical trial without prior approval from sponsor	Patients who are currently participating in an interventional clinical trial	Participation in another clinical study at the time of informed consent
	Comorbidity	Serious medical or psychiatric comorbidity	Not defined	Presence of any characteristic that may make participation inappropriate or unsafe
	Life expectancy	Less than 1 year of life expectancy	Not defined	

*No inclusion/exclusion criteria based on the stage or severity of the disease were utilized by the SUNFLOWER study, to allow inclusion of patients with symptoms ranging from mild to severe. DMP, Disease Monitoring Program; FGF23, fibroblast growth factor 23; IXLHR, International XLH Registry; SUNFLOWER, Study of Longitudinal Observation for Patients With X-linked Hypophosphatemic Rickets/Osteomalacia in Collaboration With Asian Partners; XLH, X-linked hypophosphatemia.

### 2.3 Study designs

Characteristics of the three regional studies are summarized in **Table 1**: the XLH DMP is a prospective, multicenter, longitudinal long-term outcomes program for participants with XLH (51); the IXLHR is a prospective, non-interventional observational registry (2, 52); and SUNFLOWER is a prospective, multicenter, longitudinal, long-term, observational cohort study (53, 54).

**Figure 1:**
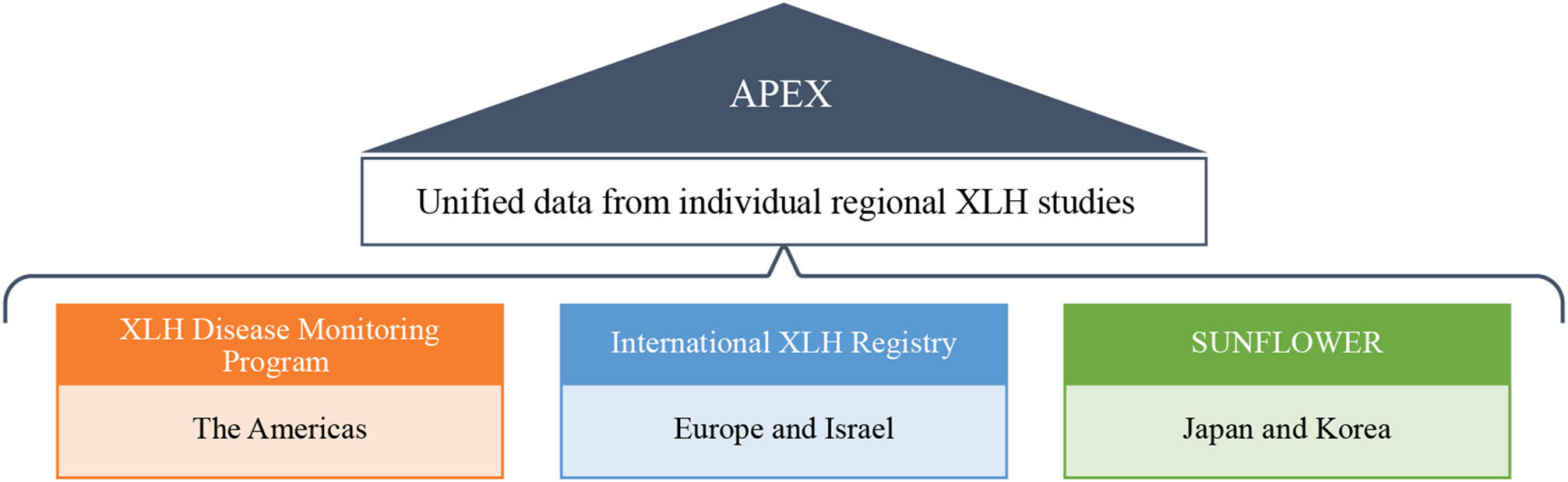
Data unification of XLH DMP, IXLHR, and SUNFLOWER in the APEX program. DMP, Disease Monitoring Program; IXLHR, International XLH Registry; SUNFLOWER, Study of Longitudinal Observation for Patients With X-linked Hypophosphatemic Rickets/Osteomalacia in Collaboration With Asian Partners; XLH, X-linked hypophosphatemia.

In all three studies, participants will be followed for up to ten years to capture treatment details and clinical outcome variables until withdrawal from the study or a loss to follow-up event. Participants will be treated at the discretion of the treating physician and will have access to burosumab only through authorized prescribed (commercial) use, or via early access programs in countries where burosumab is not commercially available. Where applicable and feasible, APEX will utilize these datasets through their unification (**Figure 1**).

### 2.4 Study objectives

The overall goals of the APEX study are to:

- Describe the progressive nature of the disorder and associated burden of patients with XLH in a global real-world setting.
- Generate data on the real-world effectiveness and safety of XLH treatment options.
- Investigate regional differences in the real-world treatment of patients with XLH.

This study was planned to analyze collected information on XLH and associated clinical, patient, and disease burden data through the long-term observation of patients with XLH. APEX will enable the exploration of regional differences and generate evidence regarding the real-world effectiveness and safety of the available therapeutic options. These data will provide further context for results from RCTs to better inform clinical decision-making, and long-term consequences of XLH or its management.

### 2.5 Determination of sample size

Due to the rarity of XLH, statistical guidance from prior research, including the three regional studies, was limited. Sample sizes of the regional studies were determined based on feasibility and the number of potential XLH outpatients who could be enrolled in participating clinical sites (2, 54). The number of participants enrolled at the regional studies is described below; however, depending on data availability, due to the status of data processing/analysis, or whether participants in the IXLHR or SUNFLOWER studies provided additional informed consent allowing for inclusion in APEX, the number of participants in the regional studies may differ from the number available for the APEX analyses. Additional consent was not required from participants in XLH DMP. At the time of submission South American data were not available for inclusion in the APEX program but may be included at a later date.

- XLH DMP: 776 patients were enrolled.
- IXLHR: 1257 patients were enrolled as of April 15, 2024.
- SUNFLOWER: 226 patients were enrolled.

### 2.6 Data collection

Detailed information on the individual regional studies’ methods of data collection is available (2, 51–54). In brief, where data are available, each study collects demographic information, medical history, *PHEX* mutation, family history, medication history, disease and medication-related complications, laboratory assessments, functional assessments, and QoL assessments. When patients enroll into their respective regional study, both retrospective and baseline data are collected. Prospective data will be collected post-baseline at routine clinical visits and added periodically to the study databases (2, 51–54).

### 2.7 Data collection tools and data management

Regional study data are collected in purposely designed electronic case report forms (eCRF) via electronic data capture tools, and study databases are checked automatically using logical checks. The eCRF is completed and signed electronically for each individual participant and is completed only by qualified individuals trained in the completion and data verification of the eCRF information. Data are stored according to local regulations in order to maintain data protection.

### 2.8 Data quality assurance

To ensure the accuracy and robustness of the collected data, the individual regional studies have mechanisms in place to assess and, if necessary, verify the data to either confirm or correct the relevant data entry(s). These include the monitoring and auditing of study sites and automatic checks built into the electronic data capture tools (2, 51–54).

### 2.9 Data unification process

Data unification and the preparation of the data for analysis are separate processes and will be performed by Arbor Research Collaborative for Health (Ann Arbor, Michigan, United States).

The data will initially undergo a data checking process, including a check on the range of the values, the units used, internal consistency and the distribution of the data. Any data flagged as impossible will be queried and cross-checked, and either corrected or removed. Following the data check, the data will undergo unification.

Data unification is an iterative process with clinical and programming review to maintain accuracy. As part of the process, a consistent naming convention for variables will be applied across the study data for variables that are identical, such as age at study entry. The data will be brought into a consistent data structure to group the data that will hold all shared values, for example baseline or longitudinal data. Finally, the data will be processed to handle inconsistent variables. This will possibly include transforming variables, such as health-related QoL patient-reported outcome values, into indicators that identify values that pass clinically meaningful thresholds. Therefore, rather than trying to translate an entire incompatible scale, established diagnostic values that identify similar concepts across the different scales can be used. The unification process will also base unification schemes on clinical input and the available literature. For example, when unifying data on permanent tooth removal where XLH DMP excludes wisdom tooth removal but the other studies do not, we may restrict comparisons to specific age ranges where, according to clinical judgment and the literature, wisdom tooth removal is likely to be rare in order to have comparable data across the individual studies.

Once the data have been unified, they will be prepared for the analysis of specific research questions. This will include handling missing data and creating clinically based categories of continuous variables. When research questions require analyses of longitudinal data, regression-based methods that are robust to differences in data collection schedules will be utilized (55). Wherever possible the full combined dataset will be used to maximize statistical power; however, region-specific analyses of variables may not be available in all studies.

### 2.10 Statistical analyses

Appropriate to an observational study, the data will be analyzed in an exploratory manner. Continuous variables will be described by standard summary statistics (e.g. number of patients, mean and standard deviation or median and inter-quartile range, as appropriate). Categorical variables will be described by frequency and proportion. Regression modeling will be used to adjust for confounding variables between comparison groups, propensity score matching will enable the identification of comparison groups to estimate the impact of an intervention in the absence of a control group, and contrasting analyses will investigate regional differences. All analyses will attempt to identify evidence of potential biases, and diagnostic tools will be used to assess goodness of fit and appropriateness of models according to accepted statistical practices. Details of all analyses will be specified in the Statistical Analysis Plan (SAP), including how missing data will be handled.

### 2.11 Management and reporting of AEs

Details of AE reports associated with any treatment for XLH will be captured by the study entry for the affected patient and included in the APEX dataset. AEs will be coded at each clinical center and reported as per the local regulatory guidelines.

### 2.12 APEX oversight

A global medical committee (GMC) has been formed with two representatives from each regional study’s scientific steering committee, and the members of the GMC may rotate with other representatives from their study. The GMC will provide scientific oversight of the APEX program, provide their input on the data collected and the analyses performed, and author APEX reports.

## 3 Discussion

Multicenter, international observational studies and patient registries can be an invaluable source of real-world data in rare disorders, providing important information that would not be possible by other means, such as RCTs. It can be challenging to derive statistically robust conclusions from RCTs in rare disorders due to the limited number of patients available for inclusion. Observational studies and patient registries, in contrast, can recruit a greater number of patients as they have fewer inclusion and exclusion criteria than RCTs and typically have a longer patient follow-up. This provides invaluable information on the natural history of a disease, treatment effectiveness, additional outcomes, and safety beyond that possible in smaller RCTs (2, 50, 56, 57). Observational studies and patient registries more closely resemble real-world clinical practice compared with RCTs, and with relatively fewer exclusion criteria, they are able to provide a more generalizable insight on the impact of certain patient characteristics on disease outcomes.

APEX is an important extension of the regional XLH studies, collecting and unifying their data to form the largest XLH patient dataset in the world to date. An advantage of creating a global dataset by pooling data and increasing the sample size is in allowing analyses to be conducted that would not normally be feasible in many rare disorders. This initiative also enables a comparison of region-specific XLH populations, treatment pathways and outcomes.

There are several areas of research in which APEX may be particularly beneficial. Firstly, there is the continuing uncertainty in the management of adult patients with XLH. Clinical practice recommendations published in 2019 recommended conventional therapy for symptomatic adults (8). However, small-scale RCTs and case studies have demonstrated benefits of burosumab treatment in adults with moderate to severe XLH (31, 32, 58, 59), but real-world evidence of treatment with burosumab is lacking in this patient group. Secondly, data from APEX may also provide invaluable information on adolescent patients with XLH transitioning into adult care, as this population can be lost to follow-up and there is uncertainty regarding optimal management (60). The absence of data from this age group occurs due to their exclusion from the burosumab RCTs (29, 30), thus real-world evidence regarding their treatment and outcomes is a significant gap in our knowledge. The large patient numbers available to the APEX project and the ability to compare regional differences in practice may provide additional evidence that can be used to further refine treatment approaches in patients with XLH, and importantly, in patient groups for whom data are lacking. Finally, the analysis of the outcomes of long-term burosumab treatment and the impact of the age of initiation on outcomes may help to identify which disease complications cannot be prevented, improved, or resolved with burosumab treatment. This will hopefully lead to additional studies to characterize and address the unmet medical needs of patients with XLH.

### 3.1 Limitations

The limitations of APEX include inconsistencies of protocol (e.g. study visit requirements) and differences in data collection and reporting methods between the individual regional studies, which may limit the data that can be pooled or used for comparison. Therefore, it may not be possible to combine all datasets within APEX. Differences in the number of patients recruited regionally, may also result in imbalances in representation, thereby affecting the ability to describe clinical practice in underrepresented regions. In addition, there are inherent limitations of non-interventional observational studies. Unlike RCTs, there is often no standardized or mandated follow-up in registries, and this can be influenced by regional differences in data collection and clinical practice. This increases the likelihood of missing or incomplete data collection and is a potential source of bias. The XLH DMP study aims to improve follow-up, with specified visit schedules and measurements funded by the study. However, these may often be carried out in conjunction with the clinical care, leading to variability in timing and collection. SUNFLOWER has specified assessments to be collected either annually in pediatric participants or every two years in adults, while IXLHR has recommended clinical variables and collection schedule, but data are collected as per routine clinical practice. While for some registries there can be a lack of supervised enrollment, the enrollment of participants in these regional studies are supervised by the study investigators based on inclusion/exclusion criteria. The clinical diagnosis is based on the local practice of expert physicians, which ensures that the regional studies accurately reflect real-world practice. However, the lack of central definitions of diseases or diagnostic practices may result in regional variability in the diagnosis and reporting of clinical conditions.

## 4 Conclusion

Observational studies and patient registries offer valuable opportunities to generate real-world data and address knowledge gaps in rare diseases. The APEX program will enable the global collection and analysis of data relating to the natural history, treatment, and outcomes of patients with XLH. Analyses may help address research questions that would not be feasible in the individual regional studies, as well as allow the comparison of regional practices and outcomes. It is anticipated the findings from APEX will further increase the understanding of XLH, its treatment, and help to improve clinical practice for patients with XLH.

## 5 Ethics and dissemination

The XLH DMP required Institutional Review Board approvals for each participating local institution. Patients who have provided informed consent, or assent where required, with informed consent by a legally authorized representative, are included in XLH DMP (51).

The IXLHR is run in accordance with the Declaration of Helsinki and received ethical, regulatory, and institutional approvals at national, regional, and site levels for each participating country. Patient data are kept in accordance with the EU General Data Protection Regulations on the processing of personal data and the protection of privacy in the electronic communication sector (2016/679/EU). Once a patient or their legal representative provides informed consent, or assent for minors aged ≥12 years, they are enrolled in the IXLHR (2, 52).

Ethics approval for SUNFLOWER was obtained from the Ethics Committee of Osaka University, the Ethics Committee of Kyowa Kirin Co., Ltd., and the Ethics Committee of each participating medical institution. Patients or their parents/guardians are required to give informed consent before inclusion in SUNFLOWER (53, 54).

Before inclusion in the APEX project, participants in the IXLHR and SUNFLOWER are provided with information regarding the objectives and the procedures and are required to reconsent to participate.

The datasets used and analyzed that support the findings of this manuscript are available from the corresponding author and Kyowa Kirin Co., Ltd. on reasonable request.
